# Perceptions of colorectal cancer screening and recommendation behaviors among physicians in Korea

**DOI:** 10.1186/s12885-017-3881-5

**Published:** 2017-12-16

**Authors:** Hye Young Shin, Mina Suh, Boyoung Park, Jae Kwan Jun, Kui Son Choi

**Affiliations:** 1 National Cancer Control Institute, National Cancer Centre, 323 Ilsan-ro, Ilsandong-gu, Goyang, 410-769 Republic of Korea; 2 Graduate School of Cancer Science and Policy, National Cancer Centre, 323, Ilsan-ro, Ilsandong-gu, Goyang-si, Gyeonggi-do 410-769 Republic of Korea; 3 College of Nursing, Korea University, 145, Anam-ro, Seongbuk-Gu, Seoul, 137-713 Republic of Korea

**Keywords:** Colorectal neoplasms, Colonoscopy, Physician, Attitude, Screening

## Abstract

**Background:**

Physician recommendations for colorectal cancer (CRC) screening have consistently been shown to be a strong predictor of screening. The aim of this study was to investigate perceptions of CRC screening modalities and recommendation behaviors among physicians in Korea.

**Methods:**

A cross-sectional, nationally representative survey conducted between November 2013 and February 2014. In total, 379 physicians (241 primary care physicians and 138 physicians affiliated with the Korean Association for the Study of Intestinal Diseases responded to this survey (overall response rate of 31.4%).

**Results:**

About 29% of all respondents “always” recommended and about 67% “sometimes” recommended CRC screening. Colonoscopy was perceived as an effective primary screening tool for CRC, and 80% of the physicians recommended colonoscopy for CRC screening. Only 7% recommended FOBT. In multivariate analysis, recommending FOBT was associated with stronger belief in the screening efficacy of FOBT (aOR 3.70, 95% CI 2.09, 6.57), weaker belief in the screening efficacy of colonoscopy (aOR 0.29, 95% CI 0.12, 0.69), and negative decisional balance for colonoscopy screening (aOR 0.82, 95% CI 0.71, 0.95).

**Conclusions:**

Although FOBT is provided free-of-charge through a nationwide CRC screening program, colonoscopy was more commonly recommended and preferred by physicians.

**Electronic supplementary material:**

The online version of this article (10.1186/s12885-017-3881-5) contains supplementary material, which is available to authorized users.

## Background

Colorectal cancer (CRC) exhibits high incidence and mortality rates across the globe [1]. In Korea, cases of CRC have increased significantly, with annual increases of 5.3% from 1999 to 2012 [2]. In 2012, CRC was one of the most burdensome diseases in Korea, with 28,988 new cases (50 per 100,000 males and 26.8 per 100,000 females) and 8135 deaths (13.7 per 100,000 males and 6.8 per 100,000 females) reported [2].

With evidence suggesting that screening reduces CRC mortality [3–6], CRC screening via fecal occult blood test (FOBT) or colonoscopy has been widely recommended [7]. Several countries have adopted use of FOBT in nationwide CRC screening programs, especially in countries where endoscopy resources are limited [7, 8]. Colonoscopy is also widely used as a gold standard to detect adenomas and CRC through visual inspection [8]. Both tests in Korea are available through either organized or opportunistic screening programs: As an organized screening program, the National Cancer Screening Program (NCSP) for CRC was introduced in 2004 for Medical Aids Program recipients and National Health Insurance Service (NHIS) beneficiaries aged 50 years or older. The NCSP provides, at no cost, an annual FOBT as the primary screening method for CRC and follow-up examinations with either colonoscopy (with biopsy if indicated) or double contrast barium enema for individuals with a positive FOBT result [9]. Apart from the organized CRC screening program, FOBT and colonoscopy testing are conducted in outpatient clinics or private health-assessment centers for opportunistic screening. However, in these cases, individuals must pay all procedure-related costs.

The success of organized screening program largely depends on how many people are involved in the program and how well providers adhere to cancer guidelines. One challenge to the success of CRC screening programs is a relatively low participation rate, compared to that for breast or cervical cancer. Globally, only half of all eligible individuals undergoes CRC screening [10]. In Korea, at the beginning of every year, target individuals are mailed invitation letters for CRC screening from the NHIS. Upon receipt thereof, individuals can visit any one of the primary clinics or hospitals designated as a CRC screening unit by the NHIS [9, 11]. Most physicians working at these CRC screening units are board-certified specialists [12]. They provide patients with a basic health behavior questionnaire and recommend them to receive FOBT. Further, physicians at CRC screening units are to recall FOBT-positive individuals and provide follow-up examinations with either colonoscopy or double contrast barium enema when needed. In the case of opportunistic screening, however, advanced and high-priced examinations are performed upon request from an individual based on their physician’s recommendation. In this context, we argue that when physicians meet directly with their patients at a clinic or hospital, their recommendations would heavily influence their patients’ decisions regarding CRC screening. However, little is known about how physicians recommend CRC screening. Indeed, only a few studies have been undertaken to investigate recommendation behaviors for CRC screening among physicians [10, 13–16], even less is known about what factors influence recommendation behaviors and the impact of physician recommendations on adherence to CRC screening.

Accordingly, we aimed to assess knowledge, beliefs, and attitudes on CRC screening via FOBT and colonoscopy among physicians in Korea. We then sought to compare differences in recommendation behaviors between primary care physicians and physicians affiliated with the Korean Association for the Study of Intestinal Diseases (KASID). Finally, we attempted to outline factors influencing physician recommendation behaviors on CRC screening and to determine how personal background factors are implicated in recommendations for CRC screening.

## Methods

### Study design and participants

We conducted a survey to investigate perceptions of and recommendation behaviors for CRC screening among physicians between November 2013 and February 2014. The survey samples were randomly selected from two representative physician groups: (1) primary care physicians who provide CRC screening through the NCSP and (2) physicians with membership to KASID. Primary care physicians were selected from a list of clinics and hospitals designated as CRC screening units by the National Health Insurance Service. Using a stratified random sampling procedure by geographical area, a total of 671 CRC screening units (30%) were randomly selected among 2206 CRC screening units. Meanwhile, from the KASID, we obtained a list of gastroenterologists and general surgeons offering colorectal cancer screening. A total of 771 KASID members were invited to participate in the survey.

The primary care physicians were surveyed by mail. Before sending out the self-report questionnaire, we contacted the primary care physicians via phone to explain the goal of the study and to identify eligible participants (*n* = 671). We excluded 134 physicians who were not directly involved in CRC screening or who refused to participate in the survey. The questionnaire and a stamped return envelope were then mailed to those who agreed to participate in the survey (*n* = 537). Next, we followed up with a phone call to those who had not responded to verify whether they received the mailed questionnaire and to encourage participation. We resent the questionnaire to those who did not receive it a second (*n* = 148) and third time (*n* = 96). For the KASID members, an online survey was administered. All physicians belonging to the KASID received a link to the online survey via email. A reward with a $10 gift card was provided for those who finished the questionnaire. In total, 379 physicians (241 primary care physicians and 138 KASID physicians) responded to this survey. The overall response rate was 31.4%. This study was approved by the Institutional Review Board of the National Cancer Center, Korea (NCC2014–0173).

### Measures

The self-report questionnaire was designed to explore each respondent’s knowledge, beliefs, and attitudes on FOBT and colonoscopy, CRC screening recommendation behaviors, and general physician characteristics (Additional file 1). Therein, six items were developed to assess knowledge of CRC screening, and were measured as a bivariate variable (correct response = 1, incorrect response = 0). For analysis, we used sums of each score for the knowledge items, with a total possible score ranging from 0 to 6. Belief in the efficacy of FOBT and colonoscopy screening was measured by the following three items: (a) belief in detecting early colorectal cancer; (b) belief in associated mortality reduction; and (c) belief in associated incidence reduction. Belief items were also rated as bivariate variables (yes = 1, no = 0), for a total possible belief score ranging from 0 (weak belief) to 3 (strong belief).

Decisional balance (“pros” and “cons”) was also measured by assessing attitudes toward FOBT and colonoscopy screening. Four items reflected positive attitudes toward the screening modalities, while another four reflected negative attitudes. All responses were assessed on a five-point Likert scale ranging from “totally disagree = 1” to “totally agree = 5.” Decisional balance score was obtained by subtracting scores for the four negative items from scores of the four positive items, resulting in a total possible score ranging from −16 (most negative attitude) to +16 (most positive attitude) for the specific tests. Positive decisional balance values reflected favorable attitudes toward screening, while negatives values reflected unfavorable attitudes.

We also examined demographic and practice data, including gender, age, year of graduation from medical school, area of specialty, and number of patients per day. Further, we requested the frequencies of their CRC recommendations and the screening modality applied to their patients.

### Statistical analysis

Descriptive statistics using Chi-square and t-test were performed to assess general physician characteristics, recommendation behaviors, and knowledge, beliefs, and attitudes on CRC screening. To investigate factors associated with physician recommendations toward a specific CRC screening test, we performed multiple logistic regression analysis, adjusting for covariates. We performed a study sample weight toward each respondent to obtain representative national estimates in statistical analyses. We performed all statistical analyses using SAS software (ver. 9.3; SAS Institute, Cary, NC, USA).

## Results

### Physician characteristics and recommendation behaviors

General characteristics and recommendation behaviors are presented in Table 1. The following statistically significant differences were noted between the primary care physicians and KASID physicians: primary care physicians were mostly male, 40 years and older, had graduated from medical school before 2000, and had a larger number of patients per day. Most KASID physicians majored in internal medicine. In regards to recommendation behaviors, KASID physicians were more likely to “always” recommend CRC screening, compared to primary care physicians (46.5% vs. 23.6%, respectively). Among those who recommend CRC screening, 79.8% of physicians recommended colonoscopy over FOBT.

**Table 1:** General characteristics and recommendations for colorectal cancer screening (Unweighted n, Weighted %)

Items	Total (*n* = 379)	Primary care physicians (*n* = 241)	KASID^a^ physicians (*n* = 138)	*P* value
General Characteristics				
Gender				
Male	320 (86.0)	215 (88.5)	105 (78.5)	0.013
Female	59 (14.0)	26 (11.5)	33 (21.5)	
Age, years				
30–39	93 (19.4)	34 (15.5)	59 (30.9)	0.003
40–49	182 (48.5)	121 (49.2)	61 (46.6)	
≥ 50	104 (32.1)	86 (35.3)	18 (22.5)	
Year of graduation, Medical school				
< 1990	100 (30.8)	81 (33.6)	19 (22.6)	0.003
1990–1999	175 (47.4)	119 (48.8)	56 (43.2)	
≥ 2000	102 (21.8)	39 (17.5)	63 (34.2)	
Specialty				
Internal medicine	324 (83.1)	195 (80.2)	129 (91.5)	0.021
General surgery	36 (10.5)	28 (11.5)	8 (7.5)	
Family medicine	19 (6.5)	18 (8.3)	1 (1.0)	
Patients per day				
< 25	38 (8.3)	13 (6.1)	25 (14.7)	< .0001
25–49	109 (26.6)	50 (21.8)	59 (40.9)	
50–99	192 (53.7)	142 (58.1)	50 (40.8)	
≥ 100	40 (11.4)	36 (14.0)	4 (3.6)	
Behaviors				
Recommendation for CRC^c^ screening				
Always	119 (29.4)	56 (23.6)	63 (46.5)	< .0001
Sometimes	249 (67.4)	176 (72.7)	73 (51.7)	
Not at all	11 (3.2)	9 (3.7)	2 (1.8)	
Recommended test for CRC screening				
FOBT^b^	27 (7.2)	21 (8.4)	6 (3.7)	< .0001
Colonoscopy	297 (79.8)	174 (75.8)	123 (91.4)	
Both tests	43 (13.0)	36 (15.9)	7 (4.9)	

KASID^a^, Korean Association for the Study of Intestinal Disease; ^b^FOBT, Fecal Occult Blood Test; ^c^ CRC, colorectal cancer

### Knowledge and beliefs on colorectal cancer screening

We assessed the physicians’ knowledge and beliefs on the efficacy of each CRC screening modality (Table 2). There were no significant differences in knowledge on CRC screening between the primary care physicians and KASID physicians (3.99 vs. 4.00, *p* = 0.934, respectively). Of the six knowledge items, one concerning colonoscopy screening interval had the lowest percentage of correct answers, while the item on primary CRC screening method in the NCSP showed the highest percentage of correct answers in both groups.

**Table 2:** Knowledge and Belief for CRC screening according to physicians

Items	Primary care physicians (*n* = 241)	KASID^§^ physicians (*n* = 138)	*P* value
Knowledge (Correct answer, Weighted %)			
CRC^¶^ screening guidelines in the NCSP^ξ^			
Starting age, years (50 yrs)	86.2	83.6	0.510
Stopping age, years (did not specify)	79.8	86.3	0.112
Initial screening method (FOBT)	99.1	96.1	0.067
FOBT^*^ screening interval (1 yr)	73.5	61.7	0.023
Colonoscopy screening			
Screening interval if there are no abnormal lesions (10 yrs)	6.7	10.7	0.183
Colonic perforation rate during colonoscopy (1 per1,000)	60.3	63.8	0.529
Knowledge scores^1)^, mean (SE)	3.99 (0.07)	4.00 (0.10)	0.934
Belief in FOBT screening efficacy (Yes, Weighted %)			
Belief in earlier stage detection	36.2	47.5	0.039
Belief in mortality reduction	34.7	47.5	0.020
Belief in incidence reduction	21.6	26.8	0.279
Belief scores^2)^ in FOBT efficacy, mean (SE)	0.91 (0.08)	1.21 (0.10)	0.015
Belief in Colonoscopy screening efficacy (Yes, Weighted %)			
Belief in earlier stage detection	97.2	96.2	0.646
Belief in mortality reduction	97.6	90.9	0.005
Belief in incidence reduction	92.5	86.1	0.062
Belief score^2)^ in Colonoscopy efficacy, mean (SE)	2.84 (0.03)	2.73 (0.05)	0.059

^1)^Knowledge scores were computed by summing the number of correct responses (correct response = 1, incorrect response = 0), with a possible range of 0–6

^2)^Belief scores were computed by summing the number of yes response for presence of belief (yes response = 1, no response = 0), with a possible range of 0–3

^ξ^NCSP, National Cancer Screening Program; ^§^KASID, Korean Association for the Study of Intestinal Disease; ^*^FOBT, Fecal Occult Blood Test; ^¶^ CRC, colorectal cancer

Regarding beliefs on screening efficacy, both primary care physicians and KASID physicians reported stronger belief in the efficacy of colonoscopy than that for FOBT. Meanwhile, KASID physicians conveyed significantly stronger belief in FOBT than primary care physicians (1.21 vs. 0.91, *p* = 0.015, respectively). For colonoscopy, although primary care physicians reported stronger belief in the efficacy thereof than KASID physicians, the difference was not statistically significant (2.84 vs. 2.73, *p* = 0.059, respectively).

### Decisional balance

The percentages of physicians who answered “totally agree” or “totally disagree” to four positive statements (pro) and four negative statements (con) for FOBT and colonoscopy are presented in Table 3. Significant differences were noted between primary care physicians and KASID physicians for all pros and cons items on FOBT and colonoscopy, with exception to one item on serious complications associated with FOBT screening.

**Table 3:** Descriptive analysis and decisional balance scores for the pros and cons of FOBT and Colonoscopy screening according to primary care physicians and KASID physicians (Weighted %)

Contents	FOBT^*^					Colonoscopy				
	Primary care physicians		KASID^§^ physicians		*P* value	Primary care physicians		KASID^§^ physicians		*P* value
	Totally agree	Totally disagree	Totally agree	Totally disagree		Totally agree	Totally disagree	Totally agree	Totally disagree	
PROS										
I am convinced that the test is effective in reducing mortality.	7.3	35.2	8.1	18.9	0.022	65.2	2.4	45.3	1.9	0.004
The test is easily recommendable without considering its cost.	47.4	10.8	35.4	8.5	0.011	8.8	16.1	2.3	3.9	< .0001
Detection of colonic polyps through the test can prevent CRC^¶^.	12.0	49.1	4.1	31.4	< .0001	79.9	1.6	61.8	2.8	0.003
People feel less psychological and physical burden, because the test does not require preparation for the test	46.4	6.3	23.0	3.9	< .0001	1.6	47.2	0.0	31.6	< .0001
CONS										
Due to a high false negative rate for the test, I feel pressure and responsibility for missing cases	62.9	1.3	36.2	1.3	< .0001	4.4	23.9	5.9	10.0	0.019
Due to a high false positive rate for the test, I am concerned about unnecessary testing and medical expenditures for additional tests	13.9	20.1	13.7	7.5	0.018	1.8	36.2	4.7	25.2	0.033
The stool collection process is embarrassing and inconvenient	13.5	16.4	16.7	4.2	0.016	–	–	–	–	–
Patients complain of inconvenience and pain caused by the procedure	–	–	–	–		18.9	8.5	7.0	3.7	0.004
I am concerned about serious complications caused by the test	2.2	92.3	0.7	95.9	0.488	4.7	14.9	2.2	6.9	< .0001
Decisional balance scores^1)^, mean (SE^ξ^)	0.77 (0.34)		0.80 (0.38)		0.949	3.54 (0.31)		3.13 (0.27)		0.320

^1)^Decisional balance scores using a 5-point Likert scale were computed by subtracting total scores for four con items from that for four pro items, with a total possible score ranging from −16 (most negative attitude to +16 (most positive attitude)

^§^KASID, Korean Association for the Study of Intestinal Disease; ^*^FOBT, Fecal Occult Blood Test; ^¶^ CRC, colorectal cancer; ^ξ^SE, standard error

Nevertheless, overall decisional balance scores, ranging from −16 (most negative attitude) to +16 (most positive attitude), were similar between primary care physicians and KASID physicians. Decisional balance scores for FOBT screening among the primary care physicians and the KSAID physicians were 0.77 and 0.80, respectively (*p* = 0.949), while those for colonoscopy screening were 3.54 and 3.13, respectively (*p* = 0.320). Notwithstanding, in both groups, decisional balance scores were lower for FOBT than for colonoscopy.

### Predictors associated with recommendations for CRC screening

Multiple logistic regression analysis was conducted to identify factors associated with physician recommendation behaviors on CRC screening (Table 4). We excluded physicians (*n* = 43) who recommended both FOBT and colonoscopy screening as an initial CRC screening modality, as we would not have been able to identify factors associated with recommendations for a specific test.

**Table 4:** Predictors associated with recommending FOBT screening (Unweighted n, Weighted %)

Characteristics	FOBT^*^ (*n* = 27)	Colonoscopy (*n* = 297)	FOBT^*^ vs. Colonoscopy
	n (%)	n (%)	aOR (95% CI)
Group			
Primary care physicians	21 (86.6)	174 (70.5)	Reference
KASID^§^ physicians	6 (13.4)	123 (29.5)	0.28 (0.07–1.14)
Gender			
Male	20 (73.0)	256 (87.9)	Reference
Female	7 (27.0)	41 (12.1)	2.82 (0.59–13.59)
Age, years			
30–39	7 (18.0)	80 (20.8)	Reference
40–49	10 (37.4)	146 (50.4)	1.14 (0.23–5.71)
≥ 50	10 (44.6)	71 (28.7)	1.19 (0.10–14.34)
*P for trend*			0.238
Year of graduation, Medical school			
< 1990	9 (37.5)	70 (28.4)	Reference
1990–1999	9 (35.2)	139 (49.1)	1.27 (0.18–9.18)
≥ 2000	9 (27.3)	86 (22.6)	1.16 (0.10–13.60)
*P for trend*			0.060
Specialty			
Internal medicine	21 (72.1)	260 (85.7)	Reference
Others	6 (27.9)	37 (14.3)	1.57 (0.35–6.98)
Patients per day			
< 25	4 (9.8)	32 (8.9)	Reference
25–49	4 (13.7)	89 (27.6)	0.48 (0.06–3.99)
50–99	15 (59.7)	144 (51.4)	0.96 (0.15–6.26)
≥ 100	4 (16.9)	32 (12.1)	1.02 (0.07–14.50)
*P for trend*			0.603
Knowledge scores, mean (SE ^ξ^)	3.77 (0.22)	4.01 (0.06)	0.57 (0.34–0.95)
Belief scores, mean (SE ^ξ^)			
FOBT	2.16 (0.19)	0.82 (0.07)	3.70 (2.09–6.57)
Colonoscopy	2.42 (0.19)	2.83 (0.03)	0.29 (0.12–0.69)
Decisional balance scores, mean (SE^ξ^)			
FOBT	4.06 (0.87)	0.48 (0.27)	1.12 (0.99–1.26)
Colonoscopy	0.20 (1.08)	3.71 (0.23)	0.82 (0.71–0.95)

^§^KASID, Korean Association for the Study of Intestinal Disease; ^*^FOBT, Fecal Occult Blood Test; ^ξ^SE, standard error

Although not statistically significant, KASID physicians were less likely to recommend FOBT screening than primary care physicians (aOR 0.28, 95% CI 0.07, 1.14). Further, physicians who exhibited higher knowledge scores on CRC screening were less likely to recommend FOBT than colonoscopy (aOR 0.57, 95% CI 0.34, 0.95). Recommendations for FOBT screening were significantly associated with a strong belief in the efficacy of FOBT screening (aOR 3.70, 95% CI 2.09, 6.57) and a weak belief in the efficacy of colonoscopy screening (aOR 0.29, 95% CI 0.12, 0.69). Similarly, decisional balance scores were also associated with recommendation behaviors: those with more favorable attitudes toward colonoscopy were less likely to recommend FOBT (aOR 0.82, 95% CI 0.71, 0.95).

## Discussion

Although the NCSP has provided CRC screening with annual FOBT for men and women aged 50 years or over since 2004, the participation rate is low at 27.6 [16]. Participation rates for CRC screening, however, could potentially be improved by motivating physicians to recommend screening [13–15]. In the current study, we found that only 29.4% of physicians “always” recommended CRC screening. Only 67.4% “sometimes” recommended CRC screening, which low compared to other countries [17, 18]. The majority of physicians recommended colonoscopy as an initial screening tool for CRC screening (79.8%), while only 7.2% of physicians recommended FOBT, although the NCSP provides FOBT at no direct cost to the patient as a primary screening test.

A high proportion of physicians recommending colonoscopy was similarly reported in previous studies [17, 18]. In Korea, colonoscopy tests are widely conducted in outpatient clinics or private screening centers, and are readily accessible. One of the reasons why physicians more frequently recommend colonoscopy than FOBT is because it is somewhat inexpensive. Compared to Western countries, the cost of colonoscopy screening is relatively cheap (approximately 80 USD), as it is partially covered by the National Health Insurance when patients have gastrointestinal health problems. Also, the availability and accessibility of colonoscopy in Korea are high: the presence of more skilled endoscopists has made mass colonoscopy quite practical. Further, Koreans tend to prefer colonoscopy test over FOBT for CRC screening. According to the Korean National Cancer Screening Survey, a higher screening rate was reported for colonoscopy than for FOBT (35.2% vs. 27.6%) [16]. While FOBT is a simple, safe, and inexpensive test with which to screen for CRC, its low sensitivity, mainly for premalignant lesions, necessitates recommendations for annual screening, with which many individuals may be reluctant to comply [19].

Interestingly, most of the physicians that participated in our survey perceived FOBT to be less effective in reducing CRC mortality and incidence, as well as in detecting early stage CRC. They reported less favorable attitudes toward FOBT performance than that for colonoscopy: for example, physicians (particular in primary care physicians) were concerned that FOBT generates a lot of false negatives and that FOBT misses a lot of cancers. They believed that colonoscopy is the more accurate technique, although it is invasive, carries risks of bleeding and perforation, requires preparation and premedication, and involves much higher costs than FOBT. This is in line with previous studies [20–24]. A study of Canadian primary physicians reported that 75.3% of physicians were concerned about false negatives for FOBT, while only 2.9% for were concerned about false negatives for colonoscopy [23]. Another study also reported that concerns for the performance of FOBT reduced recommendations thereof in CRC screening by 15% [24]. A recent study of US primary care physicians reported decreases in FOBTs performed, while colonoscopies continued to increase over the previous 3 years [22]. Nearly 86% of the physicians reported favorable attitudes toward colonoscopy as the best available test [22]. Nevertheless, FOBT has been widely adopted as a primary screening method for CRC in several countries, owing to proven performance in numerous studies [7, 8]. Of the two FOBT methods, fecal immunochemical test (FIT) has garnered higher performance ratings and higher acceptance rates than guaiac FOBT, and thus, several countries have recently posited the adoption of FIT as a primary screening tool [7, 25, 26]. In Korea, newly revised CRC guidelines recommend FIT (qualitative or quantitative) and selective use of colonoscopy, taking into consideration individual preferences and the risk of CRC [27].

In the current study, primary care physicians perceived FOBT to be less effective in reducing CRC mortality and incidence, and they had less favorable attitudes toward FOBT than colonoscopy compared with KASID physicians. However, interestingly, primary care physicians were, nevertheless, more likely to recommend FOBT than KASID physicians (8.4% vs. 3.7%, respectively). This behavior is likely associated with their daily practice: Primary care physicians in Korea provide CRC screening through the NCSP. FOBT is provided as the primary screening method at a clinic or hospital that has been designated as a CRC screening unit. Thus, primary care physicians working at designated CRC screening units can play an important role in promoting participation in and adherence with the NCSP. Also, primary care physicians tend to consider to a greater degree the costs of tests and convenience to the patient, as they likely maintain close relationships with their patients. In contrast, KASID physicians were more likely to recommend colonoscopy than primary care physicians (91.4% vs. 75.8%, respectively). Most of KASID physicians are specialists in gastroenterologist and are employed by university hospitals. Thus, they would be responsible primarily for diagnosing and treating CRC [28]. Accordingly, KASID physicians may have been more familiar with colonoscopy than FOBT, and this may have directed their focus on colonoscopy for CRC screening.

Regarding knowledge of CRC screening guidelines, over 80% of the physicians (data not shown) reported “every 5 years” as the screening interval for colonoscopy; the US Preventive Services Task Force recommends colonoscopy screening every 10 years [29]. Physician recommendations have been shown to have a positive effect on increasing perceived susceptibility to CRC and intention to be screened [14]. Under the NHIS system in Korea, there is no family medicine system. Thus, primary care physicians, especially those working at screening units, have the major task of encouraging CRC screening. While options for CRC screening allow for flexibility, they can also render decisions about recommending or choosing a particular test difficult. Each test has its tradeoffs in terms of efficacy, complications, discomfort, time, and cost. Therefore, physicians must carefully consider the messages that are communicated to the public about CRC screening. Assessments of individual screening preferences, in combination with intervention strategies to promote the performance of the preferred screening method, may increase compliance with CRC screening recommendations.

Our study findings should be interpreted in light of several limitations. First, the response rate for this survey was not high, although we encouraged participation several times using various methods. Nevertheless, primary care physicians were selected by a stratified random sampling method based on geographical area from a nationwide database, and we performed a study sample weight toward each respondent to obtain representative national estimates in statistical analyses. Second, the respondents to this survey were probably highly motivated to uphold CRC screening practices, and thus our results on CRC recommendation behaviors may be overestimated. Third, we did not consider barriers to screening recommendations from the physicians, which could provide some insight on ways to improve CRC recommendation rates and strategies that could be effective in improving CRC screening rates.

## Conclusion

Colonoscopy was found to be more commonly recommended for CRC screening than FOBT, despite the fact that FOBT is provided free-of-charge through the NCSP. The majority of physicians reported more favorable beliefs and attitudes on the test performance of colonoscopy than for FOBT. We also noted variations in CRC screening recommendation behaviors and knowledge, beliefs, and attitudes toward CRC screening (FOBT and colonoscopy) between primary care physicians and KASID members. Although colonoscopy is generally considered to have a high detection rate, it is an invasive test that must be performed by a physician. Therefore, colonoscopy tests depend heavily on the skills of the endoscopist and on the availability of a colonoscope. Even assuming that the capacity exists to perform screening colonoscopies for every age-eligible person at the recommended frequency, we recommend caution in promoting colonoscopy as a CRC screening test. Our findings suggest that the provision of balanced information and educational programs designed around evidence-based guidelines are needed to optimize clinical decision making for CRC screening.

## Additional file

Additional file 1: Questionnaire about perceptions of colorectal cancer screening and recommendation behaviors among physicians. (DOCX 26 kb)

## Abbreviations

CRC: Colorectal cancer
FOBT: Fecal Occult Blood Test
KASID: Korean Association for the Study of Intestinal Disease
NCC: National Cancer Center
NCSP: National Cancer Screening Program
