# Supplementary material for: Perceptions of colorectal cancer screening and recommendation behaviors among physicians in Korea
Source: BMC Cancer. 2017 Dec 16;17:860. doi: 10.1186/s12885-017-3881-5 (PMC5732373; doi:10.1186/s12885-017-3881-5)
Supplement: Additional file 1: — Questionnaire about perceptions of colorectal cancer screening and recommendation behaviors among physicians. (DOCX 26 kb) [file 12885_2017_3881_MOESM1_ESM.docx]

**Questionnaire about perceptions of colorectal cancer screening and recommendation behaviors among physicians**

1. The government has implemented a nationwide colorectal cancer (CRC) screening program via the National Cancer Screening Program (NCSP). Please check appropriate answers in response to the following questions regarding CRC screening.

1-1. At what age can people start receiving colorectal screening in the NCSP?

1. 40 years old ② 50 years old ③ 60 years old ④ No age in particular

1-2. What is the initial CRC screening method offered in the NCSP?

1. Fecal occult blood test (FOBT) ② Colonoscopy ③ Sigmoidoscopy ④ Nothing in particular

1-3. If no abnormalities are discovered upon CRC screening in NSCP, when should the next CRC screening be undertaken?

- 1. 6 months ② 1 year ③ 3 years ④ 5 years ⑤ 10 years ⑥ No period in particular

1-4. At what age should people no longer receive CRC screening in the NCSP?

1. 70 years old ② 75 years old ③ 80 years old ④ No age in particular

2. In the event no colon polyps or abnormalities are found via colonoscopy, what should the next colonoscopy be performed?

① 6 months ② 1 year ③ 3 years ④ 5 years ⑤ 10 years ⑥ No period in particular

3. What is the estimated rate of colonic perforation during colonoscopy?

① 1:100 ② 1:1,000 ③ 1:10,000 ④ I don’t know

4. The following questions cover beliefs about the efficacy of fecal occult blood test (FOBT) and colonoscopy screening. Please read each statement and check appropriate responses for each method.

|  | FOBT | | Colonoscopy | |
| --- | --- | --- | --- | --- |
|  | Yes | No | Yes | No |
| (1) The screening method has enough medical evidence of its capabilities to detect colorectal cancer at an early stage. |  |  |  |  |
| (2) The screening method has enough medical evidence of its capabilities to effectively reduce the mortality rate of CRC. |  |  |  |  |
| (3) The screening method has enough medical evidence of its capabilities to effectively reduce the incidence rate of CRC. |  |  |  |  |

5. Via the statements below, please indicate your opinions about conducting **FOBT** for a person at no risk of CRC. Please read and check the degree to which you agree with each of the following.

1: Totally disagree 2: Somewhat disagree 3: Partially agree 4: Somewhat agree 5: Totally agree

| (1) I am concerned about serious complications associated with the test. | Totally disagree: 1 : 2 : 3 : 4 : 5 : Totally agree |
| --- | --- |
| (2) The test is easily recommendable without considering its cost. | Totally disagree: 1 : 2 : 3 : 4 : 5 : Totally agree |
| (3) Detection of colonic polyps with the test can prevent CRC. | Totally disagree: 1 : 2 : 3 : 4 : 5 : Totally agree |
| (4) People feel less psychological and physical burden, because the test does not require preparation for the test. | Totally disagree: 1 : 2 : 3 : 4 : 5 : Totally agree |
| (5) Due to a high false negative rate associated with the test, I feel pressure and responsibility for missing cases. | Totally disagree: 1 : 2 : 3 : 4 : 5 : Totally agree |
| (6) Due to a high false positive rate associated with the test, I am concerned about unnecessary testing and medical expenditures for additional tests. | Totally disagree: 1 : 2 : 3 : 4 : 5 : Totally agree |
| (7) I am convinced that the test is effective in reducing mortality. | Totally disagree: 1 : 2 : 3 : 4 : 5 : Totally agree |
| (8) The stool collection process is embarrassing and inconvenient. | Totally disagree: 1 : 2 : 3 : 4 : 5 : Totally agree |

6. Via the statements below, please indicate your opinions about conducting a **colonoscopy** for a person at no risk of CRC. Please read and check the degree to which you agree with each of the following.

1: Totally disagree 2: Somewhat disagree 3: Partially agree 4: Somewhat agree 5: Totally agree

| (1) Due to a high false negative rate associated with the test, I feel pressure and responsibility for missing cases. | Totally disagree: 1 : 2 : 3 : 4 : 5 : Totally agree |
| --- | --- |
| (2) Due to a high false positive rate associated with the test, I am concerned about unnecessary testing and medical expenditures for additional tests. | Totally disagree: 1 : 2 : 3 : 4 : 5 : Totally agree |
| (3) I am convinced that the test is effective in reducing mortality. | Totally disagree: 1 : 2 : 3 : 4 : 5 : Totally agree |
| (4) Detection of colonic polyps with the test can prevent CRC. | Totally disagree: 1 : 2 : 3 : 4 : 5 : Totally agree |
| (5) I am concerned about serious complications associated with the test. | Totally disagree: 1 : 2 : 3 : 4 : 5 : Totally agree |
| (6) People feel less psychological and physical burden, because the test does not require preparation for the test. | Totally disagree: 1 : 2 : 3 : 4 : 5 : Totally agree |
| (7) Patients complain of inconvenience and pain caused by the procedure. | Totally disagree: 1 : 2 : 3 : 4 : 5 : Totally agree |
| (8) The test is easily recommendable without considering its cost. | Totally disagree: 1 : 2 : 3 : 4 : 5 : Totally agree |

7. The following questions concern the manner in which you recommend colorectal screening to a person at no risk of CRC.

7-1. How often do you recommend screening to others?

1. Always ② Sometimes ③ Not at all

7-2. In the event that you do, what test do you recommend for initial screening?

① FOBT ▶ **Please go to 7-3).**

② Colonoscopy ▶ **Please go to 7-4).**

③ FOBT and Colonoscopy ▶ **Please answer all (7-3, 7-4).**

7-3. If you recommend FOBT as the initial screening test, please respond to the following questions.

A. To what age group do you recommended start taking the test?

① 40 years old ② 50 years old ③ 60 years old ④ No age in particular

B. In the event test results are normal (negative), when do you recommend taking the test next?

① 6 months ② 1 year ③ 3 years ④ 5 years ⑤ 10 years ⑥ No period in particular

C. To what age group do you stop recommending the test?

① 70 years old ② 75 years old ③ 80 years old ④ No age in particular

7-4. If you recommend colonoscopy as the initial screening test, please check the following questions.

A. To what age group do you recommended start taking the test?

① 40 years old ② 50 years old ③ 60 years old ④ No age in particular

B. In the event no colon polyps or abnormalities are discovered, when do you recommend taking the test next?

① 6 months ② 1 year ③ 3 years ④ 5 years ⑤ 10 years ⑥ No period in particular

C. To what age group do you stop recommending the test?

① 70 years old ② 75 years old ③ 80 years old ④ No age in particular

◈ Please read and respond to the following questions.

1. What is your gender? ① Male ② Female

2. What is your age? [ ] years old

3. When did you graduate from medical school? [ ]

4. What is your major?

① Internal medicine ② General surgery ③ Family medicine

5. At what type of hospital do you work?

① Clinic ② Hospital ③ General hospital ④ University hospital

6. How many patients do you see per day?

① < 25 ② 25-49 ③ 50-99 ④ ≥ 100
